# Role of Bioactive Molecules on Neuroprotection, Oxidative Stress, and Neuroinflammation Modulation

**DOI:** 10.3390/ijms232415925

**Published:** 2022-12-14

**Authors:** Valentina Di Liberto, Giuseppa Mudò

**Affiliations:** Dipartimento di Biomedicina, Neuroscienze e Diagnostica Avanzata, Università di Palermo, Corso Tukory 129, 90134 Palermo, Italy

As the global population ages, the burden of neurodegenerative and neurological disorders is dramatically increasing. Despite the significant progress in the understanding of their pathogenesis, the triggers and the underlying molecular and cellular mechanisms remain debated, and some of these disorders are still incurable. Therefore, the identification of novel targets and the development of promising therapeutic strategies are real challenges for modern neuroscience.

Cholesterol homeostasis plays a major role in ensuring brain health by modulating synaptogenesis, synaptic activity, and neuronal plasticity. Defects in brain cholesterol homeostasis, although not fully elucidated, are associated with neurodegenerative disease pathogenesis [1]. Passarella et al. [2] recently reviewed the main mechanisms involved in brain cholesterol homeostasis, depending on the functioning of the “cholesterol shuttle”, responsible for cholesterol transport and distribution in neuronal and glial cells. Interestingly, the authors highlighted the existence of a cerebral bidirectional control between the cholesterol shuttle and the purinergic signaling, including the influence of purines on brain cholesterol turnover, and the rearrangement of purinergic signals caused by cholesterol shuttle alterations. In the healthy brain, purinergic signaling regulates vital neuronal and glial cell functions; however, in pathological conditions, including neurodegenerative diseases, purinergic signaling is dysregulated, leading to cell dysfunction. Given the pathological implications of the crosstalk between the cholesterol shuttle and the purinergic signaling, more in-depth research unveiling the detailed mechanisms involved in this interaction may suggest novel therapeutic targets for neurodegenerative disorders.

Alzheimer’s disease (AD) is a genetic and sporadic neurodegenerative disorder typically associated with cognitive impairment. AD pathogenesis, classically defined by the presence of β-amyloid-containing plaques and tau-containing neurofibrillary tangles, results from a complex interplay of several neurobiological factors, including oxidative stress and neuroinflammation chronicization, and aberrant microRNA (miRNA) expression. Mutations in the Presenilin-1 (PSEN1) gene are the most common cause of familial AD. PSEN1 is a crucial catalytic subunit within the γ-secretase complex, which cleaves transmembrane proteins, including the amyloid precursor protein (APP). Mutations in PSEN1 lead to β-insoluble β-amyloid (Aβ) production and/or a loss of essential PSEN functions in the brain, which in turn triggers neurodegeneration, synaptic impairment, and cognitive deficits [3]. Wuli et al. [4] have recently described that n-butylidenephthalide (BP), extracted from the Chinese herbal medicine Angelica Sinensis, which reduces amyloid plaque formation in trisomy 21 (Ts21)- induced pluripotent stem cells (iPSCs)-derived neurons, is associated with a significant reduction in PSEN1 mRNA and protein levels. Interestingly, the same treatment caused the upregulation of miR-29b-2-5p expression, and the subsequent downregulation of long noncoding RNA (lncRNA)-CYP3A43-2, PSEN1 expression, and Aβ formation. These results were further validated in vivo in 3xTg AD mice, in which the efficacy of BP in Aβ reduction was comparable to that of Donepezil, the leading drug for the treatment of AD. Altogether, these data emphasize the importance of the lncRNA (LncCYP3A43-2)–miRNA (miR29-2-5p)–mRNA (PSEN1) network in AD pathogenesis and propose miR-29b-2-5p as a therapeutic target for AD. 

Brain oxidative stress is a common pathological signature of chronic neurodegenerative and neurologic disorders. Oxidative stress occurs when the fine-tuning of reactive oxygen species (ROS) generation and elimination is impaired, leading to the overaccumulation of ROS and the oxidative damage of cellular components, the alteration of energy metabolism, and, ultimately, cell death. Considering the prominent role of oxidative stress in neurodegenerative disorders, neuroprotective antioxidants are considered a promising approach for slowing the progression and limiting the extent of neurodegeneration [5]. Recently, the antioxidant properties of natural biomolecules have been deeply investigated as an attractive tool for the treatment of neurodegenerative diseases. In this context, Nuzzo et al. [6] demonstrated that grapefruit IntegroPectin, extracted via hydrodynamic cavitation from the bio-waste resulting from citrus fruit juice extraction, exerts a powerful protective and antioxidant effect in neuronal-like SH-SY5Y neuroblastoma cells exposed to oxidant agents. Interestingly, the protective action of grapefruit IntegroPectin preserves the membrane potential and morphology of mitochondria, which are a key source and an immediate target of oxidative stress. This study underlines the potential development of this new biomolecule as an attractive therapeutic agent for oxidative-stress-associated brain disorders.

Antioxidant enzymes, involved in the transformation of ROS into stable nontoxic molecules, represent the most important endogenous defense system against oxidative-stress-induced cell damage. Peroxiredoxins (Prx) are cysteine-dependent peroxidase enzymes that play a key role in clearing cellular peroxides. A growing body of evidence has demonstrated that impaired Prx expression correlates with neurodegenerative diseases, although the role of Prx6 is less clear [7]. Recently, Turovsky et al. [8] demonstrated that exogenous Prx-6 exerts neuroprotective, antioxidant, and anti-inflammatory effects in mixed hippocampal neuroglial cell cultures during ischemia/reoxygenation, showing more selective protection towards astrocytes. Interestingly, Prx-6 exerted a more pronounced and astrocyte-directed antioxidant effect, compared with the exogenous antioxidant vitamin E. On the whole, these data suggest that the exploitation of the neuroprotective potential of Prx-6 can contribute to the development of promising novel brain-protective strategies, which can overcome the limitations associated with the delivery of exogenous antioxidants to brain cells.

Together with oxidative stress, neuroinflammation represents a major feature in the pathogenesis of chronic neurodegenerative diseases and is a key target for therapeutic interventions. The activation of glial cells and neuroinflammatory cascades leads to increased oxidative stress, which further worsens neuronal damage and neuroinflammation, resulting in a feed-forward loop of neurodegeneration [9]. In their study, Massimini et al. [10] used a murine co-culture of hippocampal neurons and inflamed microglia cells to identify a set of inflammatory marker genes linked to neurodegenerative disorders. Next-generation sequencing revealed a set of dysregulated target genes involved in the modulation of leucocyte chemotaxis, defense responses, and calcium homeostasis. Interestingly, the final selection was based on the already established contribution of identified genes to human neurodegenerative progression, and was further validated after treatment with bioactive neuroprotective molecules. The results of this study can simplify in vitro research involving neuroinflammatory phenomena, and the obtained panel of genes can be considered an easy tool for the visualization of neuroinflammatory processes and the testing of potential compounds targeting neuroinflammation.

At the beginning of the 21st century, the therapeutic management of patients affected by neurodegenerative disorders remains a major biomedical challenge. In the scenario of the intense research on neurodegenerative diseases, the information provided by this Special Issue aims to unravel novel processes underlying the pathogenesis and the progression of these disorders, suggesting new targets for the development of therapeutic strategies. 
